# The formation of multivesicular bodies in activated blastocysts is influenced by autophagy and FGF signaling in mice

**DOI:** 10.1038/srep41986

**Published:** 2017-02-03

**Authors:** Hyejin Shin, Soyoung Bang, Jiyeon Kim, Jin Hyun Jun, Haengseok Song, Hyunjung Jade Lim

**Affiliations:** 1 Department of Biomedical Science & Technology, Institute of Biomedical Science & Technology, Konkuk University, 120 Neungdong-ro, Gwangjin-gu, Seoul 05029, Korea; 2 Department of Biomedical Laboratory Science, Eulji University, 553 Sanseong-daero, Seongnam, Gyeonggi-do 13135, Korea; 3 Department of Biomedical Science, CHA University, CHA Bio Complex, 689 Sampyeong-dong, Seongnam, Gyeonggi-do 13884, Korea; 4 Department of Veterinary Medicine, School of Veterinary Medicine, Konkuk University, 120 Neungdong-ro, Gwangjin-gu, Seoul 05029, Korea

## Abstract

Dormant blastocysts during delayed implantation undergo autophagic activation, which is an adaptive response to prolonged survival in utero during less favorable environment. We observed that multivesicular bodies (MVBs) accumulate in the trophectoderm of dormant blastocysts upon activation for implantation. Since autophagosomes are shown to fuse with MVBs and efficient autophagic degradation requires functional MVBs, we examined if MVB formation in activated blastocysts are associated with protracted autophagic state during dormancy. We show here that autophagic activation during dormancy is one precondition for MVB formation in activated blastocysts. Furthermore, the blockade of FGF signaling with PD173074 partially interferes with MVB formation in these blastocysts, suggesting the involvement of FGFR signaling in this process. We believe that MVB formation in activated blastocysts after dormancy is a potential mechanism of clearing subcellular debris accumulated during prolonged autophagy.

Embryo implantation is governed by ovarian steroid hormones and their diverse downstream mediators^1^. In mice, the preovulatory estrogen surge induces intense proliferation of the uterine epithelium on day 1 of pregnancy, and this effect gradually subsides by day 3 when progesterone secretion from the newly formed corpora lutea increases. On the morning of day 4, there is a preimplantation secretion of ovarian estrogen; this is a critical requirement for embryo implantation in mice and rats. The uterus maintains a short “window” of implantation until day 4.5 of pregnancy^2^. In mice, ovariectomy (OVX) obliterates preimplantation estrogen secretion, resulting in delayed implantation and blastocyst dormancy^3^ (Fig. 1a). Such dormant blastocysts maintain a free-floating state in utero for several weeks and even months, and an estrogen injection reactivates implantation in 18–24 h^1^ (Fig. 1a). Dormant blastocysts have low metabolism, proliferate slowly, and exhibit extended longevity compared to blastocysts that are ready for implantation^4^.

Another unique feature of dormant blastocysts is that they exhibit heightened autophagic activation during delayed implantation^5^. Activation of autophagy, the self-eating process within cells^6^, seems to be an adaptive response to an unfavorable environment during prolonged survival in utero, as inhibiting autophagy reduces the survival rate of dormant blastocysts^5^. We previously observed by transmission electron microscopy (TEM) that multivesicular bodies (MVBs) accumulate in the trophectoderm of activated blastocysts that were released from the delayed implantation state by 17β-estradiol (E_2_) injection^5^. Profound accumulation of MVBs is a distinctive attribute of activated blastocysts, as MVBs are not readily observable in normal or dormant blastocysts^5^. Heavy MVB accumulation in activated rodent blastocysts is consistent with other studies^7^,^8^,^9^.

MVBs are late-stage endosomes characterized by the presence of intraluminal vesicles within a large vesicle. MVBs serve as intermediate endosomal structures that direct cargo for lysosomal degradation^10^. MVBs also fuse with autophagosomes to form amphisomes that can further fuse with lysosomes for efficient autophagic flux and target degradation^11^. When MVBs fuse with the plasma membrane, the internal vesicles containing various signaling molecules are released into the extracellular space^10^. These vesicles, usually with diameters ranging from 40–100 nm, are called exosomes and are a newly discovered mechanism of intercellular communication^12^. Exosomes carrying micro RNAs (miRNAs), proteins, and lipid mediators can be taken up by other cells to modulate various cellular responses^13^,^14^,^15^. Specific examples of exosomal functions have been established in cancer cells^13^, cells at the maternal-fetal interface^16^, reticulocytes^17^, and immune cells^18^. Microvesicle-mediated intercellular communication between embryonic stem cells and trophoblasts is also suggested as a mechanism of successful implantation^19^.

This investigation focused on determining why blastocysts activated after delayed implantation show heavy MVB formation while normal or dormant blastocysts do not. The unique characteristics of activated blastocysts is that they have gone through a prolonged period of autophagic activation during dormancy until they initiate implantation reaction^5^. Sustained autophagy for several days in dormant blastocysts can lead to the accumulation of cellular waste that needs to be removed before the blastocyst can enter a normal developmental program after implantation. A small number of MVBs are usually observable within cells, but this number increases when cells encounter any circumstance that requires the removal of cellular debris or waste. For example, reticulocytes accumulate a large number of MVBs that carry organelles and debris during maturation^20^. Experiments have also shown that efficient autophagic degradation requires functional MVBs and that a malfunction in Endosomal Sorting Complexes Required for Transport (ESCRT) machinery often leads to impaired autophagic flux, causing the accumulation of autophagosomes that do not proceed to degradation^21^,^22^,^23^,^24^,^25^. Thus, we hypothesized that the conspicuous MVB accumulation in the trophectoderm of activated blastocysts is associated with prolonged autophagic activation in dormant blastocysts during delayed implantation. To test this hypothesis, we used a combination of approaches including an *Atg5* deficient mouse model. We herein showed that MVB formation in activated blastocysts is a result of sustained autophagy in dormant blastocysts and suggested that fibroblast growth factor (FGF) receptor signaling is a potential mediator of MVB formation.

## Results

### Dormant blastocysts resuming implantation show MVB accumulation in trophectodermal cells

**Figure 1:**
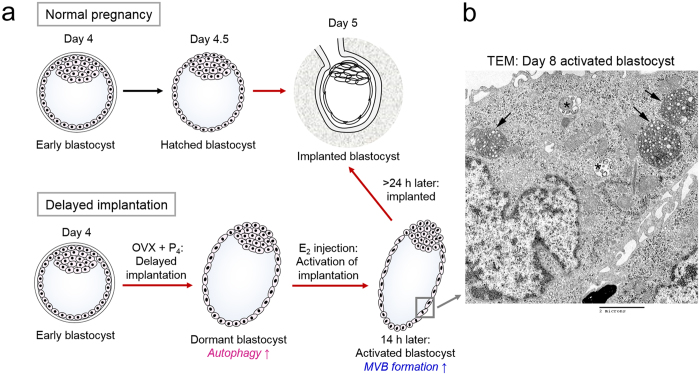
Mouse model of implantation and delayed implantation. (**a**) Mouse embryos escape the zona pellucida on day 4.5 of pregnancy and implant. In mice, implantation requires ovarian estrogen secreted on the morning of day 4. Ovariectomy (OVX) on the morning of day 4 of pregnancy removes the source of estrogen and thus delays implantation. Pregnancy was maintained by daily injections of progesterone (P_4_) but blastocysts enter a dormant state (“dormant blastocyst”). These embryos live longer than normal blastocysts and exhibit heightened autophagic activation^5^. When an injection of 17β-estradiol (E_2_) is given, dormant blastocysts are activated and implantation is initiated (“activated blastocyst”). We previously showed that dormant blastocysts exhibit heightened autophagy, and activated blastocysts accumulate MVB in the trophectoderm. (**b**) Transmission electron microscopy analysis of a day 8 activated blastocyst. A trophectoderm cell is shown at 3000X. Arrows, multivesicular bodies (MVB); asterisks, late endosomes.

**Figure 2:**
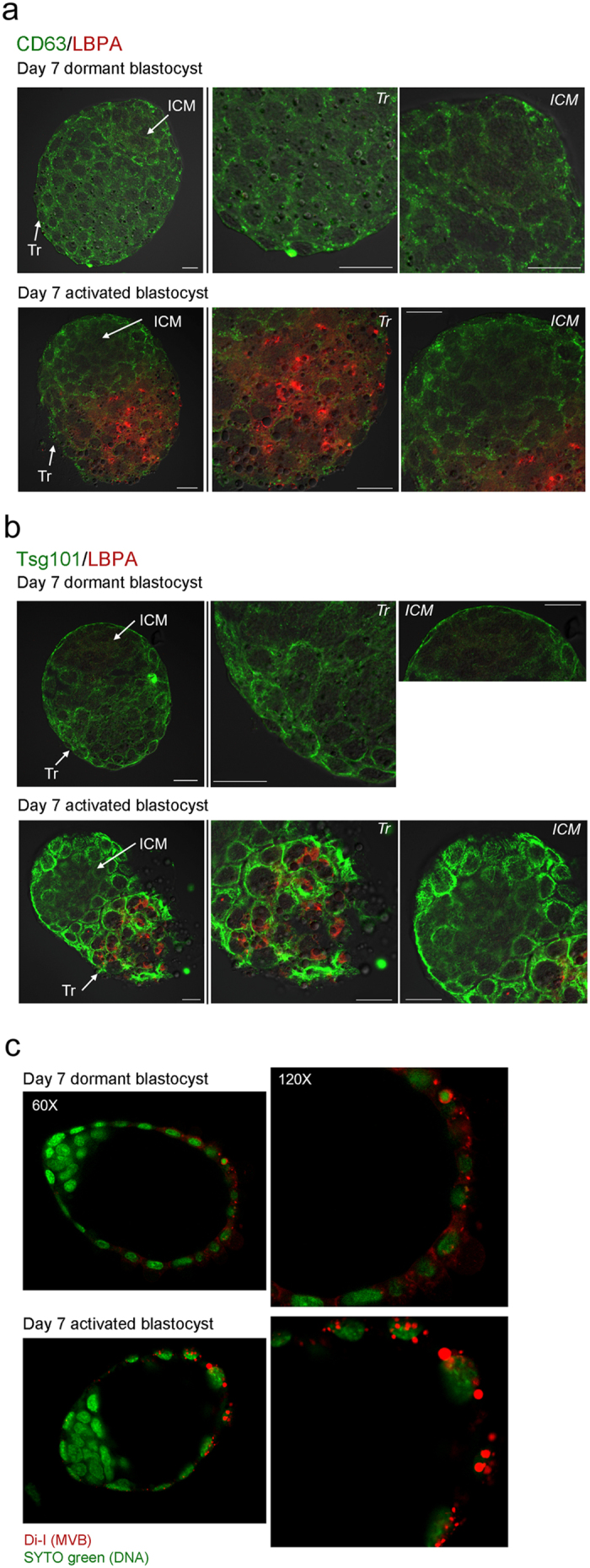
Numerous MVBs form in the trophectoderm of activated blastocysts. (**a**) Immunofluorescence staining of CD63 and LBPA in dormant and activated blastocysts. Day 7 dormant and activated blastocysts were obtained and subjected to immunofluorescence staining. In both dormant and activated blastocysts, CD63 (green) exhibited a uniform puncta pattern throughout the trophectoderm (Tr). In contrast, LBPA (red) accumulation prominently increased in the mural trophectoderm of activated blastocysts where the blastocyst makes the initial contact with the endometrium during implantation. LBPA was not observed in the inner cell mass (ICM). Mouse or rabbit IgG was used as a mock control (data not shown). CD63 staining was repeated three times in 16 dormant (4 mice) and 20 activated blastocysts (5 mice) with similar results. Scale bar, 20 μm. (**b**) Immunofluorescence staining of Tsg101 and LBPA in day 7 dormant and activated blastocysts. Compared to the basal level of Tsg101 expression (green) in dormant blastocysts, Tsg101 accumulation dramatically increased in the trophectoderm of activated blastocysts. LBPA accumulation (red) in the mural trophectoderm of activated blastocysts was again confirmed. Mouse or rabbit IgG was used as a mock control (data not shown). LBPA staining was repeated three times in 17 dormant (4 mice) and 15 activated blastocysts (4 mice) with similar results. Tsg101 staining was repeated twice in 15 dormant (4 mice) and 12 activated blastocysts (3 mice) with similar results. Scale bar, 20 μm. (**c**) Confocal live imaging of Di-I stained embryos. Day 7 dormant and activated blastocysts were stained with dye (red) to stain internal vesicles of MVBs^31^. Nuclei were counterstained with SYTO 11 green fluorescence nucleic acid stain. Numerous Di-I-positive puncta are shown in the trophectoderm of activated blastocysts. The experiment was repeated three times with 28 dormant and 23 activated blastocysts.

During our previous TEM analysis to investigate autophagic vacuole formation in dormant and activated blastocysts^5^, we observed that numerous MVBs form in trophectodermal cells of activated blastocysts (Fig. 1b). To first determine whether these MVBs are the conventional late endosomal structures reported in diverse cell types, we examined the expression of widely used MVB markers in activated blastocysts. MVB biogenesis depends on a dynamic network of the ESCRT complexes 0, I, II, and III^26^. Tsg101, a crucial component of the ESCRT-I complex, is often used as a MVB marker^27^. CD63, a tetraspanin molecule, is also often used as a marker of MVBs^28^,^29^, but it generally exhibits a wider distribution than ESCRT components^28^. The function of CD63 is not yet clear with respect to MVB formation and exosomal release^10^. Lysobisphosphatidic acid (LBPA) is a unique lipid species in the inner vesicles of MVBs^30^. Di-I is a live dye that labels membrane lipids and was used to track small vesicles secreted by mouse embryos^31^. As shown in Fig. 2a, CD63 is widely expressed in both dormant and activated blastocysts. In contrast, Tsg101 and LBPA accumulation increased in activated blastocysts, but the LBPA signal was confined to the mural trophectoderm (Tr) where the blastocyst makes initial contact with the uterine epithelium during implantation (Fig. 2b). Di-I live imaging also showed that the number of Di-I-positive small vesicular structures was increased in the mural trophectoderm of activated blastocysts (Fig. 2c). All the increased signals except CD63 were observed in the Tr and are clearly negative in the inner cell mass (ICM).

### Normally implanting blastocysts do not accumulate LBPA

**Figure 3:**
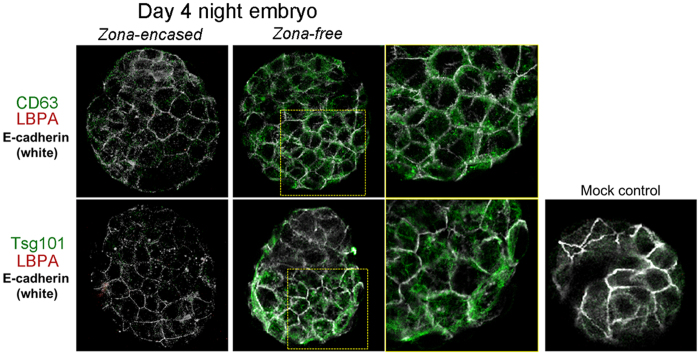
Expression of MVB markers in day 4 late blastocysts. Immunofluorescence staining of MVB markers in day 4 blastocysts. Blastocysts were obtained at 9–10 PM on day 4. At this time, most blastocysts have escaped from the zona pellucidae (zona-free) and are ready for implantation, while some stay within them (zona-encased). Blastocysts were stained with the indicated antibodies. To stain the trophectodermal cell border, an anti-E-cadherin antibody was used^57^. In zona-encased blastocysts, CD63, Tsg101, and LBPA were expressed at very low levels. In zona-escaped blastocysts, CD63 (green) and Tsg101 (green) were upregulated, but LBPA (red) was not observed. The experiments were repeated three times in average 6 zona-encased and 14 zona-free blastocysts for each antigen.

Our previous TEM analysis showed that day 4 blastocysts near the time of implantation do not show noticeable MVB formation^5^. Thus, we next examined the expression of CD63, Tsg101, and LBPA in normally implanting blastocysts to see which marker expression reflects ultrastructural analyses. We collected blastocysts on the night of day 4 (around 9–10 PM), near the time of implantation when most blastocysts are zona-free. Some blastocysts still retain their zona pellucidae; however, zona-free blastocysts are considered closer to implantation. As shown in Fig. 3, regardless of the presence of the zona pellucida, these blastocysts show very low or undetectable LBPA signals. Expression of Tsg101 and CD63 increased in zona-escaped blastocysts. Negative LBPA staining in day 4 night blastocysts was consistent with our previous report the absence of distinct MVBs at this stage by TEM^5^. These results showed that the increased expression of Tsg101 and CD63 in day 4 late blastocysts is not linked to the physical accumulation of MVBs, whereas LBPA accumulation reflects MVB status in activated blastocysts. Therefore, subsequent experiments used LBPA as a marker for MVBs.

### MVB formation in blastocysts undergoing activation coincides with ubiquitin accumulation

**Figure 4:**
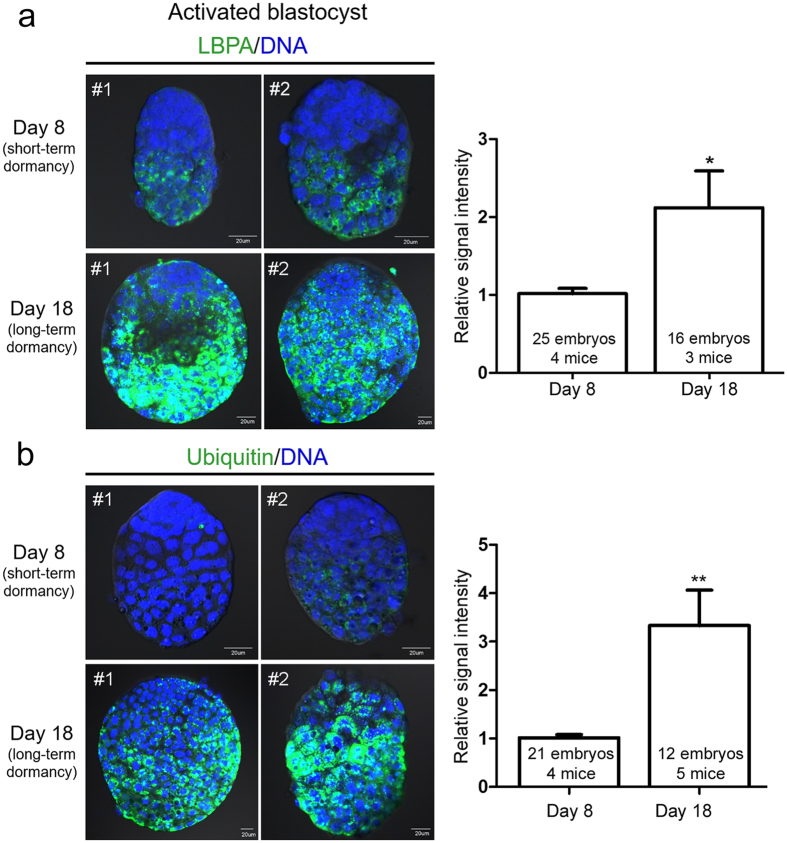
Accumulation of ubiquitin and LBPA increases in activated blastocysts after long-term delayed implantation. Immunofluorescence staining was performed in short-term activated (activated on day 8) and long-term activated blastocysts (activated on day 18). (**a**) Immunofluorescence staining of LBPA was performed in indicated numbers of blastocysts for each group. Four independent experiments were performed and three sets showing similar results were included in statistical analysis (see Methods). Signal intensities were obtained by setting the whole blastocyst as the region of interest. The mean of values obtained for day 8 activated blastocysts in each experiment was normalized to 1. Two representative embryos (#1 and #2) are shown for each group. Nuclei were counterstained with TO-PRO-3 Iodide stain (blue). Scale bar, 20 μm. *p < 0.01. (**b**) Immunofluorescence staining of ubiquitin was performed in indicated numbers of blastocysts for each group. Four independent experiments were performed and were included in statistical analysis. Signal intensities were obtained by setting the whole blastocyst as the region of interest. The mean of values obtained for day 8 activated blastocysts in each experiment was normalized to 1. Both LBPA and ubiquitin signals were significantly higher in day 18 activated blastocysts. Two representative embryos (#1 and #2) are shown for each group. Nuclei were counterstained with TO-PRO-3 Iodide stain (blue). Scale bar, 20 μm. **p < 0.001.

Considering that only activated blastocysts show heavy MVB accumulation, it is possible that heightened autophagy during preceding dormancy is the main cause of this phenomenon. This link is clear in many systems because the formation of numerous MVBs can be observed in cells with upregulated autophagy whereas autophagic vacuoles accumulate when MVB formation is blocked (reviewed in refs 24 and 32). By directly interacting with ubiquitin, some ESCRT components help recruit ubiquitinated proteins to MVBs for efficient degradation^33^,^34^. Ubiquitination also serves as a sorting signal for targeted autophagy-mediated degradation of various proteins^35^. Thus, we hypothesized that high MVB formation in activated blastocysts occurs to remove old or unusable ubiquitinated proteins. To examine if there is any correlation between MVB formation and ubiquitin accumulation, we compared LBPA expression and ubiquitin levels in day 8 (short-term dormancy) and day 18 (long-term dormancy) activated blastocysts. As shown in Fig. 4, ubiquitin accumulation was hardly observable in day 8 activated blastocysts. Day 18 activated blastocysts showed a dramatic accumulation of ubiquitin accompanied by an augmented LBPA signal. This coordinated increase in LBPA and ubiquitin in the long-term dormancy group suggested that MVBs carry out a degradative function to clear up autophagic waste. It is possible that the longer the period of autophagic activation, the greater the cellular waste accumulation.

### LBPA accumulation depends on autophagic activation during dormancy

**Figure 5:**
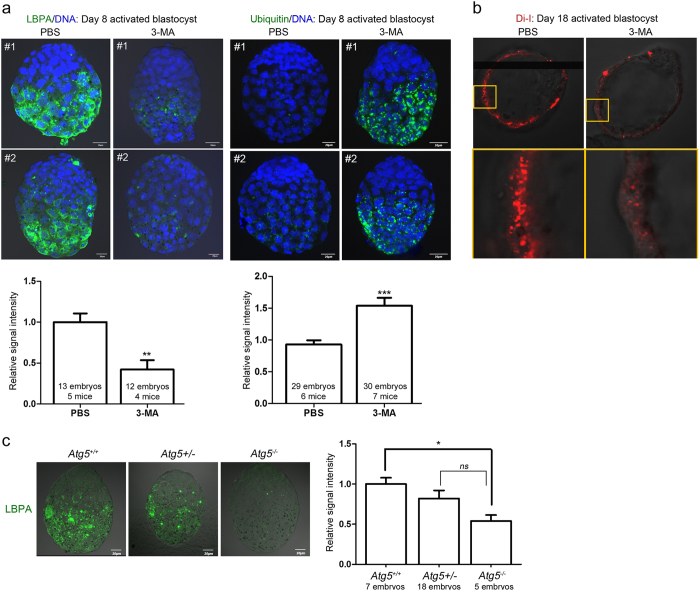
MVB formation is associated autophagic activation in activated blastocysts. To inhibit autophagy, 3-MA (5 mM in 100 μl PBS) injections were given daily at 9 AM to delayed implanting mice from the day of OVX to the day of E_2_ injection. (**a**) Immunofluorescence staining of LBPA or ubiquitin in day 8 activated blastocysts from PBS- or 3-MA-injected delayed implanting mice. Immunofluorescence staining was performed in indicated numbers of blastocysts for each group. For LBPA staining, four independent experiments were performed and three sets showing similar results were included in statistical analysis. For ubiquitin staining, seven independent experiments were performed and four sets showing similar results were included in statistical analysis. Signal intensities were obtained by setting the whole blastocyst as the region of interest. The mean of values obtained for blastocysts from PBS-injected mice in each experiment was normalized to 1. For each image, five Z-series sections of 1 μm (LBPA) or four sections of 2 μm (ubiquitin) were stacked. Scale bar, 20 μm. **p < 0.001; ***p < 0.0001. (**b**) Di-I confocal live imaging of day 18 activated blastocysts obtained from PBS- or 3-MA-injected mice. DiI-positive puncta were observed in the trophectoderm of activated blastocysts. 3-MA injection to block autophagy during delayed implantation led to a reduced number of DiI-positive puncta. Enlarged images of yellow-boxed areas are shown in the lower panel. This experiment was repeated 6 times (16 activated blastocysts from PBS-injected mice [n = 6] and 19 activated blastocysts from 3-MA-injected mice [n = 6]). (**c**) LBPA immunofluorescence staining in *Atg5*^+/+^, *Atg5*^+/−^, and *Atg5*^−/−^ activated blastocysts. *Atg5*^+/−^ female mice were bred with *Atg5*^+/−^ male mice and ovariectomy was performed on day 4 of pregnancy. On day 8 of delayed implantation, mice received an E_2_ injection to activate implantation and all embryos were collected 14 h later and subjected to LBPA immunofluorescence staining. See Methods section for detailed information. Scale bar, 20 μm. *p < 0.01; ns, not significant.

We next tested whether autophagic activation is a prerequisite for MVB formation. To accomplish this, we used a pharmacological inhibitor of autophagy, 3-methyladenine (3-MA)^36^, which we have previously used in this model of delayed implantation^5^. 3-MA was injected (5 mM, intraperitoneal injections) to delayed implanting mice daily until they received an E_2_ injection on day 8 or 18. Activated blastocysts were collected 14 h later. As shown in Fig. 5a, 3-MA injections to short-term activated blastocysts (day 8) led to decreased LBPA accumulation in trophectoderm of activated blastocysts. Inversely, 3-MA-treated activated blastocysts showed increased ubiquitin accumulation (Fig. 5a), suggesting that inhibiting autophagy leads to poor MVB formation and inefficient removal of ubiquitin-tagged proteins. In the long-term activated blastocysts (day 18), 3-MA injection also decreased the number of Di-I-positive puncta in the Tr (Fig. 5b). These results indicated that autophagic activation during dormancy is a prerequisite for the formation of numerous MVBs in activated blastocysts.

Next, we further confirmed the link between autophagy and MVB formation using *Atg5* knockout (KO) mice. Atg5 is a major upstream factor of the autophagic cascade, and deletion of this gene leads to lethality in newborn mice^37^. Delayed implanting *Atg5*^+/−^ female mice were activated with E_2_ injection on day 8, and all activated blastocysts were subjected to LBPA immunofluorescence staining. Each embryo was genotyped afterwards. As shown in Fig. 5c, *Atg5*^−/−^ blastocysts show almost undetectable LBPA signals compared to *Atg5*^+/+^ blastocysts. *Atg5*^+/−^ blastocysts showed a subtle reduction in LBPA signals. Collectively, these results clearly demonstrate that prolonged autophagic activation during dormancy is a precondition for MVB formation at the activation of implantation.

### FGFR signaling modulates MVB formation in blastocysts undergoing activation

**Figure 6:**
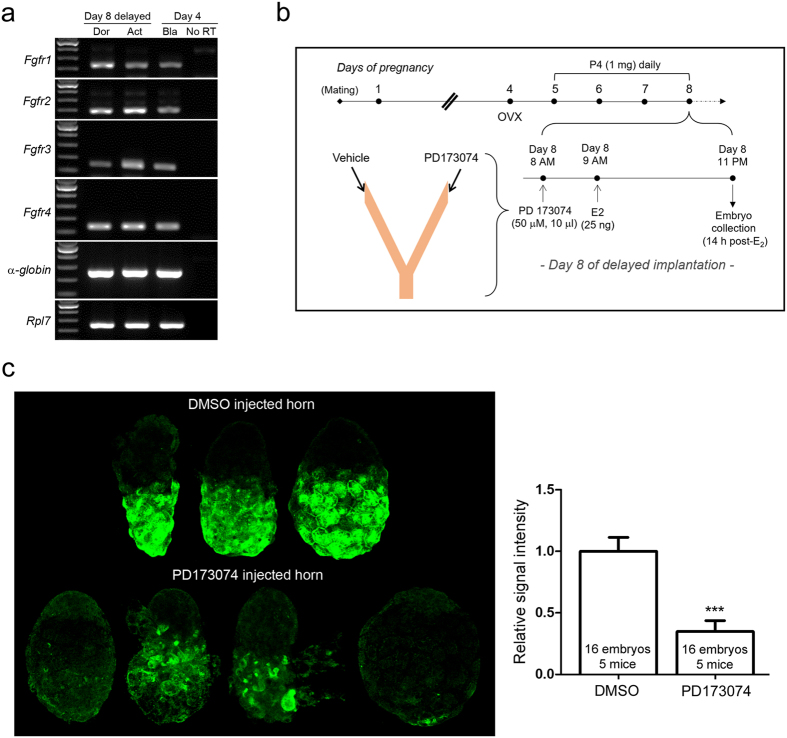
The involvement of FGF signaling in MVB biogenesis of activated blastocysts after delayed implantation. (**a**) mRNA expression of FGFRs in normal, dormant, and activated blastocysts. Day 4 normal blastocysts were obtained from day 4 pregnant mice by uterine flushing. Dormant and activated blastocysts were obtained from day 8 delayed implanting mice. In each group, 10 embryos were pooled and subjected to RNA extraction, reverse transcription, and PCR with specific primers for mouse FGFRs (Table 1). All these receptors were expressed in day 4 normal, day 8 dormant, and day 8 activated blastocysts. (**b**) The experimental scheme. Ovariectomy was performed on mice on the morning of pregnancy day 4 and mice subsequently received P_4_ injection from day 5 to 8. On day 8, 1 h prior to P_4_ and E_2_ injections, a small volume of PD173074 (50 μM in DMSO) was injected into one uterine horn. The volume was less than 5 μl. The other horn was injected with same volume of 0.25% DMSO. Hormones were injected 1 h later to ensure that blastocyst activation did not occur before PD173074 injection. Activated blastocysts were obtained 14 h post-E_2_ injection and subjected to LBPA immunofluorescence staining. (**c**) Expression of LBPA in activated blastocysts collected from PD- or vehicle-injected horns. The experiment was performed in 8 mice. A dramatic reduction of LBPA (green) accumulation was noted in 16 embryos for each group that were collected from 5 mice. These data were included for statistical analysis as shown. Signal intensities were obtained by setting the whole blastocyst as the region of interest. The mean of values obtained for embryos collected from DMSO-injected horns for each experiment was normalized to 1. A set of embryos from one mouse is shown. For each image, five Z-series sections (1 μm) were stacked. Scale bar, 20 μm. ***p < 0.0001.

We next sought to identify a candidate signaling molecule that promotes MVB formation in blastocysts. Fibroblast growth factors (FGFs) are often found in exosomal cargo^10^ and induce vesicular trafficking in the node of postimplantation embryo^31^. Additionally, FGFs are modulators of autophagy in various cellular systems^38^,^39^. Fibroblast growth factors (FGFs) also play important roles in preimplantation and postimplantation embryonic development^40^. Four types of FGF receptors (FGFRs), FGFR1–4, are all expressed in early mouse embryos at different intensities^41^. FGFR2 works with FGF2 in trophectodermal cells during blastocyst expansion^42^, whereas FGFR3 and FGFR4 are expressed in all blastocyst cells^41^,^43^. FGF4 is also a crucial factor in trophoblast stem cell maintenance^44^. We first confirmed that mRNAs for all FGFRs are present in day 8 dormant, activated, and day 4 blastocysts (Fig. 6a). To examine whether perturbation of FGFR signaling with an inhibitor has any influence on MVB formation in activated blastocysts, we devised the experimental scheme shown in Fig. 6b. PD173074, an FGFR inhibitor, was injected into one horn of the lumen of delayed implanting mice 1 h prior to E_2_ injection. The other horn was injected with same volume of vehicle. Following PD173074 and E_2_ injections, activated blastocysts were obtained 14 h post-E_2_ injection and subjected to LBPA immunofluorescence staining to assess the degree of MVB formation. Compared to embryos collected from vehicle-injected horns, activated blastocysts from PD173074-injected horns exhibited decreased LBPA accumulation (Fig. 6c), suggesting that FGFR signaling affects the dramatic accumulation of MVB in the Tr upon activation of implantation.

## Discussion

The highlight of this investigation is that heightened autophagy in dormant blastocysts results in MVB accumulation in blastocysts triggered to be activated. We provide evidence for this observation with multiple approaches, combining the use of pharmacological and genetic tools to manipulate autophagy. *Atg5*^−/−^ or 3-MA-exposed embryos showed very low or undetectable LBPA signals after activating of implantation, suggesting compromised MVB formation. One function of degradative MVBs is to remove cellular waste tagged with ubiquitin^45^. 3-MA injection led to reduced LBPA signals accompanied by increased ubiquitin, suggesting a functional link between MVB formation and waste removal. It is interesting to note that normally implanting blastocysts at day 4 night do not show increased MVB formation^5^. Considering the lack of LBPA accumulation in day 4 zona-free blastocysts (Fig. 3), it is prudent to conclude that MVB formation may not be a part of the normal implantation process. Among the markers tested, LBPA localization best represent MVB formation in activated blastocysts because it clearly matches with our previous TEM analysis^5^. The wider distribution of Tsg101 than LBPA observed within trophectodermal cells of activated blastocysts is also in accordance with results of a previous study^27^. Unlike LBPA, expression of both CD63 and Tsg101 increased in zona-free day 4 blastocysts. Whether CD63 and Tsg101 are involved in other cellular functions during implantation warrants further investigation. On note, Tsg101 protein has a versatile nature; roles for this protein have been reported regarding cytokinesis and viral budding^46^.

In our previous study, we showed that prolonged autophagic activation (long-term dormancy), is associated with poor developmental outcome^5^. When developmental activation is reinitiated with estrogen, dormant blastocysts implant and develop further. However, the developmental competence as assessed by fetoplacental weight and morphology clearly demonstrate a poor outcome for long-term dormant blastocysts^5^. As we showed herein, blastocysts activated after long-term dormancy (Fig. 4) showed significantly higher levels of LBPA and ubiquitin. We present a hypothesis that inefficient removal or too much accumulation of ubiquitin beyond the cellular capacity is linked to poor developmental competence of the long-term dormancy blastocysts we observed previously.

What is the biological implication of the augmented presentation of MVBs in the Tr during the activation of implantation? ESCRT proteins and MVBs are implicated in the attenuation of various signaling pathways mediated by membrane receptors^47^,^48^. Dormant and activated blastocysts have arrays of receptors that are dynamically regulated depending on the state of dormancy^49^,^50^. One such receptor, the insulin-like growth factor 2 receptor (Igf2r), is expressed in the Tr of dormant blastocysts but is rapidly downregulated in activated blastocysts^50^. Igf2r is also known as the cation-independent mannose 6-phosphate receptor (CI-M6PR), and functions to attenuate IGF signaling^51^. In the context of embryo development and implantation, insulin-like growth factor 2 (IGF2) is an important mediator of embryonic growth and viability^52^,^53^. Thus, it is possible that sustained Igf2r expression in dormant blastocysts is responsible for slow growth during dormancy whereas its downregulation upon implantation activation is required for normal developmental process to continue^50^. One potential role for MVBs could be to downregulate such signaling receptors^48^. Whether certain signaling factors are indeed modulated by MVB-mediated trafficking requires further investigation.

We had previously observed using TEM that after delayed implantation, small vesicles exit from trophectodermal cells in some activated blastocysts^5^. These small vesicles exiting from trophectodermal cells may be the exosomes resulting from the fusion of MVBs and the plasma membrane. This observation is reminiscent of the phenomenon that occurs at the postimplantation embryonic node where small vesicles exit under the regulation of FGFR signaling^31^. This resemblance led us to determine the involvement of FGFR signaling in our activated blastocyst system. PD173074 is a widely used inhibitor of FGFR signaling and was previously used in the mouse uterus^54^. Since all four FGFRs are expressed in dormant and activated blastocysts, our experiment cannot discern which FGF or receptor is responsible for the observed result. FGFR signaling is complex and requires multiple factors for signaling^55^; further experiments are required to elucidate the specific factors involved in MVB formation.

## Methods

### Materials

Primary antibodies used were as follows: rabbit polyclonal anti-Tsg101 (#ab30871, Abcam, Cambridge, MA, USA), rabbit polyclonal anti-CD63 (#sc-15363, Santa Cruz Biotechnology, CA, USA), mouse monoclonal anti-ubiquitin (#MK-11-3, MBL, Woburn, MA, USA), mouse monoclonal anti-LBPA (#Z-PLBPA, Echelon, Salt Lake City, UT, USA), and rat monoclonal anti-E-cadherin (#U3254, Sigma-Aldrich, St. Louis, MO, USA). Secondary antibodies used for immunofluorescence staining are as follows: chick anti-rabbit Alexa Fluor 488, donkey anti-mouse Alexa Fluor 568, and goat anti-rat Alexa Fluor 647 antibodies (Invitrogen, Carlsbad, CA, USA). To counterstain nuclei, TO-PRO-3-iodide (Invitrogen) or SYTO 11 green fluorescent nucleic acid stain (Invitrogen) was used. For confocal live imaging of MVBs, 1,1′-Dioctadecyl-3,3,3′,3′ -tetramethylindocarbocyanine iodide (Di-I) (AS-84711; ANASPEC, Fremont, CA, USA) (absorption/emission at 549/565 nm). PD173074, a FGFR antagonist, was purchased from Selleckchem (S-1264, Houston, TX, USA), and wortmannin (W-1628) and and 3-MA (M-9281) were purchased from Sigma-Aldrich.

### Mice

All mice were maintained in accordance with the policies of the Konkuk University Institutional Animal Care and Use Committee (IACUC). This study was approved by the Konkuk University IACUC (approval number KU14149). Five-week-old virgin CD-1 female and male mice were purchased from Orient-Bio (Gyunggi-do, Korea). *Atg5* knockout (KO) mice^37^ were obtained from the RIKEN BioResource Center (Ibaraki, Japan). *Atg5* KO mice were backcrossed to ICR for more than 6 generations to improve general reproductive performance. Mice were sacrificed under anesthesia (2.5% avertin) to minimize suffering.

### Delayed implantation and collection of embryos

To induce delayed implantation, pregnant mice were ovariectomized at around 8 AM on day 4 of pregnancy and injected daily with P_4_ (2 mg/0.1 ml sesame oil, subcutaneous injection) from day 5 until a day before sacrifice. Dormant blastocysts were collected from these mice on the indicated day. To resume implantation, P_4_-primed delayed implanting mice were injected with estradiol-17β (E_2_, 25 ng/0.1 ml oil, subcutaneous injection) in addition to P_4_. Activated blastocysts were obtained 13–14 h after E_2_ injection. Dormant or activated blastocysts were recovered by flushing uteri with M2 medium (Sigma-Aldrich). Figure 1 depicts the mouse model of delayed implantation.

### Transmission electron microscopy

Embryos were fixed with 2.5% glutaraldehyde (Sigma-Aldrich) in PBS (pH 7.2) for 2 h at room temperature and then washed with PBS. Detailed procedure of transmission electron microscopic analysis is described elsewhere^5^.

### Immunofluorescence staining

Embryos were placed onto silane-coated glass slides (Muto Pure Chemicals Co., Tokyo, Japan) by a brief centrifugation. They were fixed in 3.7% formaldehyde in PBS and permeabilized with 0.25% Triton X-100 in PBS. They were then incubated with 2% BSA in PBS for 1 h followed by incubation with specific primary antibodies at 2–4 μg/ml in 2% BSA in PBS for 2 h or overnight. After washing, secondary antibodies were applied for 40 min, followed by TO-PRO-3-iodide staining (1:250) for 20 min. Finally, the embryos were mounted using Prolong Gold Antifade reagent (Invitrogen). Slides were examined using an Olympus Fluoview FV1000 Confocal Microscope (Tokyo, Japan) equipped with multi Argon-ion (457, 488, and 515 nm), He-Ne (green, 543 nm), and He-Ne (red, 633 nm) lasers. Images were analyzed using Fluoview software version 1.5 (Olympus). In some experiments, Z-series sectional images with a thickness of 1–2 μm were serially obtained and compiled (4 or 5 sections). Embryos from delayed implanting *Atg5*^+/−^ mice were stained and then scraped from the slides for individual genotyping.

### Confocal live imaging

For live imaging of DiI-positive vesicles, unfixed embryos were placed in KSOM media (Millipore, Billerica, MA, USA) containing 5 μM of Di-I and 1 μM of SYTO 11 green fluorescent nucleic acid stain for 30 min in a 37 °C incubator under 5% CO_2_. Embryos were placed on a glass-bottom plate (SPL Lifesciences, Pocheon, Korea) and observed under a confocal microscope equipped with a warm plate. Images were obtained for 10-20 min at 20 sec intervals (2 μm sections).

### Inhibition of autophagy

3-methyladenine (3-MA, 100 μl of 5 mM solution in PBS) was injected intraperitoneally daily to delayed implanting mice from day 5 to the day of activation (the day of E_2_ injection).

### Delayed implantation in Atg5 knockout mice

*Atg5*^+/−^ female mice were bred with *Atg5*^+/−^ male mice and ovariectomy was performed on day 4 of pregnancy. On day 8 of delayed implantation, mice received an E_2_ injection to activate implantation and all embryos were collected 14 h later and subjected to LBPA immunofluorescence staining. Each embryo was numbered, scraped from the glass slide, and subjected to genotyping. Total 30 activated blastocysts from 6 *Atg5*^+/−^ female mice were successfully genotyped (*Atg5*^+/+^:*Atg5*^+/−^:*Atg5*^−/−^ = 7:18:5). Signal intensities were obtained by setting the whole blastocyst as the region of interest. The mean of values obtained for *Atg5*^+/+^ blastocysts in each experiment was normalized to 1.

### Intraluminal injection of FGFR antagonist during delayed implantation

PD173074 was purchased from Selleckchem (Houston, TX, USA) and dissolved in dimethyl sulfoxide (DMSO). Approximately 5 μl of PD173074 solution (50 μM) was delivered into one uterine horn of day 8 delayed implanting mice at 8 AM using a mouth-controlled micropipette. The other uterine horn received vehicle only (0.25% DMSO in PBS) as a control. One hour later, E_2_ (25 ng/0.1 ml) was injected to reinitiate implantation. Activated blastocysts were prepared 13–14 h later and subjected to immunofluorescence staining.

### RT-PCR

Total RNA was isolated from dormant, activated, or day 4 blastocysts (10 blastocysts/group) using TRIzol Reagent (Life Technologies, Invitrogen) according to the manufacturer’s protocol. Rabbit α-globin RNA (10 pmol/sample, Sigma-Aldrich) was used as an external RNA control. The general RT-PCR procedure has been described previously^56^. Ribosomal protein L7 (*Rpl7*) was compared as an internal control. Primers used in RT-PCR are shown in Table 1^ ^41.

### Statistical analysis

Signal intensities of immunofluorescence staining were acquired by Fluoview program by setting the entire blastocyst as region of interest (ROI). Statistical evaluation was performed on GraphPad Prism softeware (version 5.01, La Jolla, CA, USA). Unpaired Student’s t-tests were used for statistical evaluation and *p* < 0.05 was considered statistically significant.

## Additional Information

**How to cite this article:** Shin, H. *et al*. The formation of multivesicular bodies in activated blastocysts is influenced by autophagy and FGF signaling in mice. *Sci. Rep.* **7**, 41986; doi: 10.1038/srep41986 (2017).

**Publisher's note:** Springer Nature remains neutral with regard to jurisdictional claims in published maps and institutional affiliations.

## Figures and Tables

**Table 1:** Primers used for RT-PCR.

Gene name		Sequence (5′-3′)	Product size	GenBank Accession No.
*Fgfr1*	F	GAA GAC TGC TGG AGT TAA TAC CA	234 bp	NM_010206.3
	R	CAG GAG ATC AGG AAG GCC CC		
*Fgfr2*	F	AGG GAT TGC TGG CAT GCT GT	195 bp	NM_201601.2
	R	GTC TGG AGA AAA CAC AGA ATC GTC		
*Fgfr3*	F	GGA CCC TAG CCC GCC CTG CTA C	146 bp	NM_001163215.2
	R	ACT CTA CAG CAG TGC ATG TTC CC		
*Fgfr4*	F	TTT CTA GTT CCC CCA AAC AAC CTA G	133 bp	NM_008011.2
	R	ACA CCA GAG CTG ATG CCC CTT T		
*rpl7*	F	TCA ATG GAG TAA GCC CAA AG	246 bp	NM_011291.5
	R	CAA GAG ACC GAG CAA TCA AG		
Rabbit *α-globin*	F	GCA GCC ACG GTG GCG AGT AT	257 bp	NM_001082389.2
	R	GTG GGA CAG GAG CTT GAA AT		

F, forward; R, reverse.
