# Corrigendum: The formation of multivesicular bodies in activated blastocysts is influenced by autophagy and FGF signaling in mice

**DOI:** 10.1038/srep44973

**Published:** 2017-03-28

**Authors:** Hyejin Shin, Soyoung Bang, Jiyeon Kim, Jin Hyun Jun, Haengseok Song, Hyunjung Jade Lim

Scientific Reports 7: Article number: 4198610.1038/srep41986; published online: 02 03 2017; updated: 03 28 2017

The Acknowledgments section of this Article contains a typographical error, where:

“This study was supported by Konkuk University in 2015”.

should read:

“This study was supported by Konkuk University in 2014”.

